# Preliminary validation of two math screening tools to identify gifted students in grades 3–5 in Jordan

**DOI:** 10.3389/fpsyg.2025.1478767

**Published:** 2025-02-06

**Authors:** Bashir Abu-Hamour, Hanan Al Hmouz

**Affiliations:** ^1^College of Humanities and Social Sciences, Zayed University, Abu Dhabi, United Arab Emirates; ^2^Sharjah Education Academy (SEA), Sharjah, United Arab Emirates

**Keywords:** gifted students, Arabiya tests, math curriculum-based-measurement, scales validation, inclusive education

## Abstract

**Introduction:**

Early screening for mathematically gifted students (MGSs) in Jordan and other Arab countries is limited, posing challenges in identifying and providing appropriate educational services. This study evaluates the validity and reliability of the Arabiya Calculation Test and Math Curriculum-Based Measurement (M-CBM) as tools for effectively screening and supporting MGSs in inclusive education settings. These tools were developed based on the Jordanian curriculum and international assessment tools and require further validation for use in other Arabic-speaking countries.

**Methods:**

A quantitative research design was employed, using the Arabiya Calculation Test and M-CBM to assess 78 MGSs in grades 3, 4, and 5 across three schools in Jordan. The tools’ reliability and validity were evaluated, with findings specifically limited to these grades. Performance differences among students and correlations between the two measures were analyzed.

**Results:**

The findings demonstrated that both the Arabiya Calculation Test and M-CBM are valid and reliable tools for identifying MGSs. These tools effectively differentiated performance across grades 3, 4, and 5. In addition, the significant correlation between these two measures supported their validity in identifying gifted students.

**Discussion:**

The results have important implications for educational practice and policy in Jordan and similar Arab countries. Accurate identification of gifted students may facilitate tailored instruction and enrichment programs, improving the experience of inclusive education. These assessment tools offer the potential to identify gifted students early and meet their needs within an inclusive school environment.

## Introduction

Early childhood is considered a critical period of growth and development for gifted children. During this time, children’s brains continue to develop, making early intervention likely to have the most significant impact (Ansari and Pianta, 2024). Through early identification and intervention, we can accelerate the growth of young children who may be gifted (Smith, 2021). Recurring themes and findings from the literature provide a strong rationale for an increased focus on the needs of young children showing signs of potential. Numerous authors underscore the importance of early educational intervention for gifted children, arguing that gifted education should emulate special education by recognizing individualized needs as early as possible to provide responsive instructional environments, allowing potential to be actualized (Porter, 2020). Some children develop observable gifts and talents in areas such as spoken language/linguistics, reading, and mathematics distinguishing them from their same-age peers who follow a more common developmental trajectory. Other researchers (e.g., McGowan et al., 2016) suggested a more specific definition as “gifted individuals are those who demonstrate outstanding levels of aptitude (defined as an exceptional ability to reason and learn) or competence (documented performance or achievement in top 10%) in one or more domains. Domains include any structured area of activity with its own symbol system (e.g., mathematics, music, language) and/or set of sensorimotor skills (e.g., painting, dance, sports).

As a case in point, mathematical giftedness continues to be a topic of great interest in educational and psychological research, as educators and policymakers seek effective strategies to identify and support exceptionally talented individuals in mathematics. Although advancements in cognitive science have shed light on the cognitive characteristics of mathematically gifted individuals and have concluded that gifted students have above-average intelligence, as evidenced by their heightened working memory capacity, processing speed, advanced logical reasoning, and exceptional problem-solving skills (e.g., Calderón-Tena, 2016), researchers on mathematical giftedness (Wallace et al., 2018) emphasize the need for a multi-dimensional approach to identification. Research has explored the limitations of relying solely on IQ tests and has advocated for the inclusion of achievement assessments, teacher recommendations, performance-based evaluations, and Math Curriculum-Based-Measurement to capture the full range of mathematical abilities (Johnsen and VanTassel-Baska, 2020).

Evidence suggests that failure to recognize and nurture these early talents can result in negative emotional and social consequences such as masking behaviors, code-switching and possible long-term underachievement (Abu-Hamour and Al Hmouz, 2013). Researchers (e.g., Borland, 2009) estimate that approximately 3 to 5% of the school-age population are gifted students. Comparable prevalence has been suggested in Jordan and other Arab countries as well (AlGhawi, 2017; Al-Hroub, 2023). In view of this fact, it is of critical importance to conduct accurate screening assessment to identify gifted students then use the results to provide them with appropriate services. Assessment is a systematic process of collecting data that can be used to make decisions about students (Brookhart and Nitko, 2019). We assess students to learn what we need to do to serve their needs. We also assess students to determine if what we are doing is effective. Fortunately, several decades of research consistently point to strong relations between the performance of children with special needs (e.g., gifted students) and other standardized achievement measures (e.g., Arabiya Achievement Tests, Abu-Hamour and Al Hmouz, 2022a; Woodcock-Johnson IV Tests of Achievement, Schrank et al., 2014) and Curriculum-Based-Measurements (CBMs) (Salvia et al., 2017). Researchers have suggested that both types of assessments provide valuable information in the field of special and inclusive education (Abu-Hamour et al., 2013b; Mather and Abu-Hamour, 2013). Specifically, and for the purpose of this study, the Arabiya Calculation Test (standardized assessment) and Math-CBM (M-CBM) were developed to provide a quick and easy method to measure computation performance that would be both valid and reliable, and could be used to screen for gifted students in mathematics in Arab countries.

## Arabiya achievement tests and CBM

The Arabiya Achievement Tests (Abu-Hamour and Al Hmouz, 2022a) are part of the family of the Woodcock–Johnson Cognitive and Achievement Tests (WJ IV; Schrank et al., 2014) and were standardized and normed in Arabic (see *https://riversideinsights.com/arabiya-intelligence-achievement* for further details). The Arabiya Achievement Tests are based on the Jordanian norms that were established for individuals ranging in age from 4 years to 90 years. According to the Arabiya Achievement Tests manual (Abu-Hamour and Al Hmouz, 2022b), the Arabiya Calculation Test may be used with confidence to accurately screen, diagnose, and monitor progress in mathematics achievement for gifted students. In addition, private and public schools can use the test to: (a) screen for gifted students and determine their eligibility for receiving extra services; (b) set performance goals to determine the level of student’s progress required by the end of the semester or academic year; (c) compare the school’s overall results with other national or international schools; and (d) evaluate the effectiveness of the gifted education program based on the students’ progress.

CBM is considered a type of authentic assessment practice designed to provide prevention and intervention services to students (January and Klingbeil, 2020). CBM is a set of standardized procedures that were initially designed to index the level and rate of student achievement within the basic skill areas of reading, mathematics, written expression, and spelling (Alqahtani, 2024; Deno, 2003; January and Klingbeil, 2020). Researchers indicate that CBM can provide accurate information about a student’s academic standing and progress, which can then be used for a variety of psychoeducational decisions that include: (a) identifying students for special services such as gifted education (Marston, 2012; McGowan et al., 2016); (b) formulating goals and objectives for Individualized Educational Plans (IEPs; Mather and Abu-Hamour, 2013); (c) monitoring students’ progress and improving educational programs (Fuchs et al., 2024); (d) transitioning students to less restrictive environments (Hosp et al., 2016); (e) predicting how well students will perform on statewide competency tests of achievement (Buck and Torgesen, 2018); and (f) using it as an alternative assessment procedure for monitoring progress and guiding the selection of interventions (Fuchs et al., 2024; January and Klingbeil, 2020; Hosp et al., 2016).

## Psychometric properties of Arabiya achievement tests and CBM

Regarding the reliability, for tests such as Arabiya Achievement Tests, reliability coefficients must approximate or exceed 0.80 in magnitude, but coefficients of 0.90 or above are considered the most desirable (Salvia et al., 2017). Abu-Hamour and Al Hmouz (2022b) reported in the manual of Arabiya Achievement Tests three types of reliabilities: test–retest reliability, split-half procedure for nontimed tests, and Rasch reliability for the timed test. The median reliabilities ranged from 0.87 to 0.93 for the individual tests and 0.96 for the full achievement scale. Regarding validity, solid evidence was provided to support content validity, developmental patterns, concurrent validity, and predictive validity. For example, the hierarchical multiple regression analyses revealed that the best model of predicting students’ GPA across grades 1 to 12 consisted of all Arabiya Achievement Tests with a higher contribution from Test 1: Calculation, while R2 for the model = 0.44, and adjusted R2 = 0.43 (Abu-Hamour et al., 2015). In addition, it was documented in the manual that there were statistically significant differences between gifted students and average students on all of the Arabiya Tests, with the gifted students obtaining higher scores.

The validity and reliability of CBMs are well established in USA (National Center on Response to Intervention, 2010). Several studies have also confirmed the validity and reliability of the CBM in Arabic countries (e.g., Abu-Hamour et al., 2013a; Alqahtani, 2024). In addition, data from commercially available CBM, CBM manuals, and research studies provide compelling evidence that CBM develops in a linear fashion from first to eighth grade (e.g., AIMSweb; www.aimsweb.com, 2024; Hosp et al., 2016; Keller-Margulis et al., 2014). In terms of the predictive validity, several studies (e.g., Nelson-Strouts et al., 2020) have examined the relationship between CBM and statewide standardized achievement tests, especially in reading. These studies found the correlation between performance on a measure of oral reading fluency taken at the end of third or fourth grade and performance on the state assessments to range between 0.44 (Washington) and 0.79 (Illinois). On average, most studies reported correlations in the 0.60 to 0.75 range (Hosp et al., 2016). Few studies have reported outcomes of relationships between CBM and statewide assessments in math. For example, Helwig et al. (2002) examined the effectiveness of a M-CBM concept task at predicting eighth-grade student scores on a computer adaptive test of math achievement designed to approximate a state (Oregon) standardized math achievement measure. Results indicated that the M-CBM task used in this study was effective at predicting scores on the computer adapted test of math assessment for students in general education. In fact, when the data were analyzed using discriminant function analysis, the M-CBM probes predicted with 87% accuracy the students who would meet the state math standards. Helwig et al. (2002) noted that assessments such as M-CBM that can accurately estimate progress toward statewide goals in addition to monitoring classroom progress have considerable utility for planning instruction. Another study (Shapiro et al., 2006) was conducted to examine the relationships among reading, math computation, and math concepts/applications CBMs and the statewide standardized achievement test as well as published norm-referenced achievement tests in two districts in Pennsylvania. Results showed that CBM had moderate to strong correlations with midyear assessments in reading and mathematics and both types of standardized tests across school districts. Furthermore, some researchers suggested that CBM can be one source of data that could be used to potentially identify those students likely to be successful or fail the statewide assessment measure (Mather and Abu-Hamour, 2013).

## Arabiya calculation test and M-CBM

Brief standardized assessments and CBMs are widely used for the universal screening of academic skills. Universal screening programs assess all students in a population (e.g., classroom, school, or district) with the intent of identifying those who are not making sufficient progress (e.g., students with dual exceptionalities) or those who are making significant progress (e.g., gifted students) compared to their grade-level peers. The Arabiya Calculation Test and M-CBM can serve as effective screening tools if they successfully differentiate students based on their abilities. Considering factors such as validity, reliability, assessment time, and sensitivity to differences is essential when selecting universal screening measures. Many schools have found these two measures to be valuable screening tools (Abu-Hamour, 2018; Abu-Hamour and Al Hmouz, 2022b). The M-CBMs have been developed for three areas: early numeracy, computation, and concepts and applications. Computation has been the traditional standard of the M-CBM and, therefore, has the most research supporting its use (Deno, 2003). The computation CBMs were designed to offer a quick and reliable method for assessing computational skills that correlate with outcome measures. For the purposes of this study, the term ‘M-CBM’ specifically refers to computational skills. Finally, M-CBM uses percentile ranks to represent students’ performance, while the Arabiya Calculation Test provides a range of scores, including standard scores, percentile ranks, age and grade equivalents, and relative proficiency indexes, for various educational purposes (Abu-Hamour and Al Hmouz, 2022b).

## Context of the study

In Jordan and other Arab countries (e.g., United Arab Emirates), gifted students benefited from acceleration and/or enrichment programs. There are specialized schools and centers for gifted students that provide them with these services. In addition, the Ministry of Education (MoE) urges all schools to build a gifted and talented policy for their students with special focus on: (a) the identification process of the gifted and talented students, (b) the in-class and extra-curricular provision programs and resources needed, and (c) the evaluation and monitoring of gifted and talented students. In terms of the eligibility criteria, gifted students must have special gifts or talents in one or more of the following areas: (a) intellectual ability; (b) subject-specific aptitude (e.g., science, mathematics, language), (c) social maturity and leadership, (d) visual and performing arts (e.g., art, theatre, recitation), or (e) psychomotor ability (e.g., distinguished performance in one or more sports) (Ministry of Education, 2019). With regard to the actual practice, practitioners in the Jordan and other Arab countries have noted that assessments, identification, and provisions for the gifted and talented students studying in public and private schools are not provided and the trend in Arab countries has been reluctance to screen for early signs for gifted students in very young children. This was confirmed by the number of gifted students who received services across the Arab countries. For example, although the population of Jordan was estimated to be approximately 9,531,712 (Jordan Statistical Yearbook, 2017), only 3,250 gifted students were served in three gifted education schools, 18 pioneer centers, and 24 resource rooms (Ministry of Education, 2019). In conclusion, Jordan and other Arab countries are still behind in identification and service delivery to gifted students.

## Significance of the study

Universal screening data enable decisions about referrals or talent pool designation in gifted identification assessment. Students who require a referral will need additional tests or information about achievement, performance, and/or other characteristics. Students identified for a talent pool will require further differentiated instruction and experiences with monitoring of progress over time to decide when and if a formal referral and comprehensive body of evidence is required for identification. Teachers in the inclusive education era are accountable for providing differentiated instruction for gifted students. The decision regarding what type of differentiated instruction (e.g., flexible cluster grouping by topic or mathematics achievement, enrichment experiences, and increased use of technology) is necessary should be based upon an evaluation. Teachers must add components to each lesson and modify the content for their high-ability students. For example, a lesson on calculating the area of polygons might include just the basic formula for most students but should provide various real-world applications of calculating area for gifted learners (January et al., 2016).

Though empirically validated means for assessing “rapid rate of learning” in gifted populations remain unclear, both standardized achievement tests and CBM have been used as developmentally and ecologically sensitive measures to reliably and validly assess student’s response to instruction and intervention in the general population (Hixson et al., 2014). As compared to reading, not as much is known about the use of a standardized achievement test and the CBM, and math performance for gifted students. Reading and literacy are often considered the most important skills taught in schools; however, many argue that math is similarly important for life success. For gifted students, a strong foundation in math not only enhances problem-solving skills but also fosters critical thinking and creativity, which are essential for excelling in advanced academic and professional fields. To the best of the authors’ knowledge, previous work has not explored the use of the Arabiya Calculation Test and M-CBM as screening tools to identify gifted students in math.

## Objectives

The objectives of the current study are three-fold: (a) assess the reliability of the Arabiya Calculation Test and M-CBM as measures of math skills among MGSs in grades 3, 4, and 5; (b) determine whether MGSs demonstrate different performances on the Arabiya Calculation Test and M-CBM in grades 3, 4, and 5; and (c) examine the correlation between the performance of MGSs on the Arabiya Calculation Test and M-CBM in grades 3, 4, and 5 of this study.

## Method

### Participants

The sample consisted of 78 gifted students from third, fourth, and fifth grades (26 gifted students for each grade). These students were divided into three subsamples according to the grade. Half of the participants were female, and another half were male. The father’s level of education for all of the participants was a bachelor’s degree and above. Based on the previous research (e.g., McGowan et al., 2016), the researchers used the following criteria for participant selection: (a) must have had documented achievement in top 10% in math for two semesters before conducting the current study; (b) must have a Math GPA of 90% and above in one semester before conducting the current study; (c) must be nominated by both the classroom teacher and the math teacher as a gifted student in mathematics; and (d) must have a documented intelligence assessment with an Intelligence Quotient (IQ) of 117 or above for the purpose of this research. Participants have been administered the Woodcock-Johnson IV Tests of Cognitive Abilities (WJIV; Schrank et al., 2014) one year before conducting this research (2018/2019). According to the students’ profiles in the schools, participants of this research have an IQ range of 118 to 127, and the highest performance areas were documented in quantitative knowledge, processing speed, visual processing, and fluid reasoning. In addition, participants have been identified as gifted students in Math, and provided enrichment activities within the differentiated instructions practice in the schools.

All participants were selected based on the study criteria and consent forms were sent to parents seeking their agreement of participation. Parents who agreed to let their children participate in the study were requested to complete a short questionnaire that explained the inclusion criteria of this study. The participants were selected from a larger set of students (159) who were assessed to meet the requirements for inclusion in the study: gifted in math, native speakers of Arabic, no noted emotional or behavioral disorders, no noted attention disorders, and no sensory impairments. The mean ages of the participants were 99, 112, and 123 months for third, fourth, and fifth grades, respectively, with a range of 97–126 months. These students were enrolled in the first semester of the 2019/2020 school year. All participants were administered the Arabiya Calculation Test and the M-CBM probes. Participants were recruited from three private schools in the central region of Jordan. The data collection was completed by two trained teachers under the supervision of the authors. Both of the teachers had degrees in math education and childhood education.

## Measures

### Arabiya calculation test

Arabiya Calculation Test is a one test from the Arabiya Achievement Battery (Arabiya Tests; Abu-Hamour and Al Hmouz, 2022a, 2022b). Arabiya Tests are part of the family of the Woodcock–Johnson Cognitive and Achievement Tests (WJ IV; Schrank et al., 2014). The Arabiya Tests are based on Jordanian norms for individuals ranging in age from 4 years to 90 years (for further details, see: https://riversideinsights.com/arabiya-intelligence-achievement). The Arabiya Calculation Test has a median split-half reliability of 0.87, and positive findings were reported in the manual regarding the content validity, discriminant validity, concurrent validity, and predictive validity. The Arabiya Calculation Test is a test of math achievement measuring the ability to perform mathematical computations. The items in the test require the examinee to perform addition, subtraction, multiplication, division, and combination of these basic operations, as well as some geometric, trigonometric, logarithmic, and calculus operations. The calculations involve negative numbers, percents, decimals, fractions, and whole numbers.

The Arabiya Calculation Test was carefully adapted and standardized for the Jordanian context, ensuring cultural and curricular relevance. The test incorporates content aligned with the local curriculum, including mathematical concepts and operations commonly taught in Jordanian schools, such as addition, subtraction, multiplication, division, percentages, and various levels of equations. The test items and scoring procedures were designed to align with the educational expectations and learning outcomes specified by Jordan’s Ministry of Education and the WJ IV Tests of Achievement (ACH). This alignment, along with differences between the norms of the WJ IV Tests of Achievement-Calculation Test and the Arabiya Calculation Test, resulted in two tests that are similar but not identical. Because the calculations are presented in a traditional problem format in the subject response booklet, the student is not required to make any decisions about what operations to use or what data to include. The test was individually administered and took approximately 10 min for each student. The standard score was used for the test–retest reliability based on a recommendation from the Arabiya Achievement Test Manual, while the raw score was used for the rest of the statistical analyses.

### M-CBM computation

**Figure 1:**
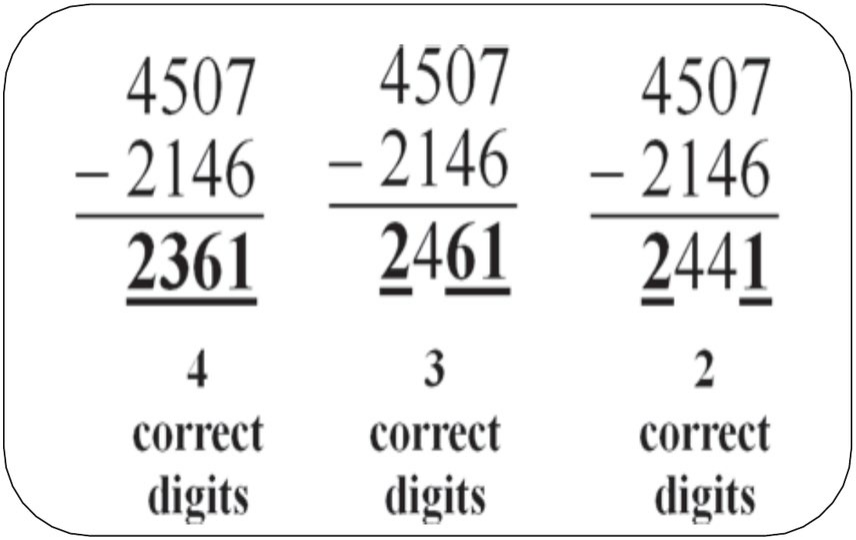
Example of M-CBM probe.

M-CBM computation probes can be administered individually or to groups of students. For the purpose of this study, we administered them individually and used multiple-skill worksheets that covered the computational skills for the targeted grades. The gifted students were given the worksheet and then asked to complete as many items as possible within two minutes. The M-CBM assigned credit to each individual correct digit appearing in the solution to a math fact (see Figure 1). By separately scoring each digit in the answer of a computation problem, the examiner is better able to recognize and give partial credit to a student. The probes were scored according to the correct digit system in this research. Three equivalent Math CBM probes were individually administered to each participant in this study. Similar to the international practice (e.g., Hosp et.al, 2016), the median score of these three probes were used to provide the most valid representation of the student’s performance. The M-CBM probes were contextualized by designing multiple-skill worksheets to align with the Jordanian math curriculum for grades 3, 4, and 5. The scoring methods and administration instructions adhered to internationally validated practices while ensuring relevance to local educational standards and the unique characteristics of the Arabic language. These adaptations were reviewed by educational assessment professionals and math educators in Jordan, who provided feedback to refine the tools for optimal linguistic and academic alignment.

### The math GPA

The Math GPA reflects a student’s ability on math computational skills in the accredited Arabic curriculum in Jordan. The Math GPA is a numeric sum of all tests achieved in classes at a given school semester. The purpose of GPA is to provide a barometer as to overall performance of a student in his or her classes, as well as create a system that allows for comparisons between students, and a class ranking system. In the Jordanian educational system, students are ordered and assigned a numerical rank against their peers based on their GPA, starting with number 100 for the student with the highest GPA and 0 for students with the lowest GPA. The rubric for the Arabic GPA is excellent (90–100), very good (80–89), good (70–79), satisfactory (60–69), minimal pass (50–59), and failure (< 50). In this research, the mean Math GPA was 93.56 for all participants with a range of 90 to 99 and standard deviation (SD) of 3.39. The means were 94.56 (SD = 3.81), 93.44 (SD = 3.21), 92.66 (SD = 3.01) for third, fourth, and fifth grades, respectively.

## Procedures

The selected three private schools were approached to coordinate the study work with the principals and teachers at the beginning of the academic semester. Each participant was provided with a small gift (e.g., toy or notebook) to encourage them to be part of the study. Incentives were used in this study to have a higher response rate for participation and increase the level of performance. As a preliminary step of this study, the authors provided four hours of intensive training for the two teachers on the administration of the Arabiya Calculation Test and M-CBM. In addition, a research assistant who has a master’s degree in educational psychology was trained to supervise the quality assurance (e.g., reliability and fidelity) of the research. The Arabiya Calculation Test and M-CBM were administered in one testing session (approximately eighteen minutes) to save set-up time and obtain accurate scores. Fidelity of administration and interrater reliability of scoring fidelity ranged from 99 to 100%.

## Data analysis

The Statistical Package for the Social Sciences (SPSS), version 24, was used to analyse the data. Descriptive statistics (means, standard deviations), Pearson product moment correlations, and one-way independent Analysis of Variance (ANOVA) were used to investigate the three study questions (Field, 2024). Regarding content validity, items of the two study measures and the specification tables were provided to eight referees in the field of educational assessment and math education who work in four universities in Jordan and the Ministry of Education to judge the content of the Tests, the administration procedures, and the format. The referees highly recommended the use of the study measures to identify gifted students with some minor suggestions related to the language phrasing of the administration instructions and the format of the M-CBM (e.g., enlarging the font).

## Results

### Preliminary data analysis

**Figure 2:**
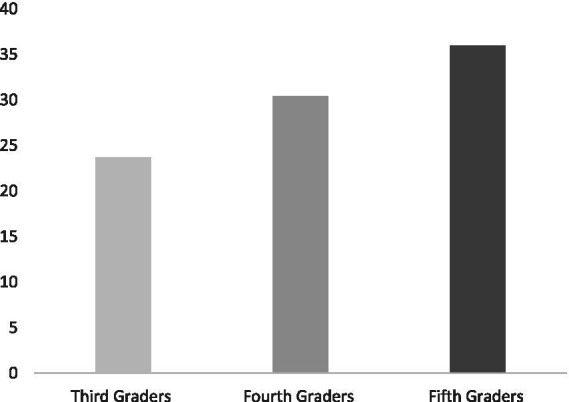
Graphic display of the mean performance on the Arabiya calculation test reported in number of correct answers.

**Figure 3:**
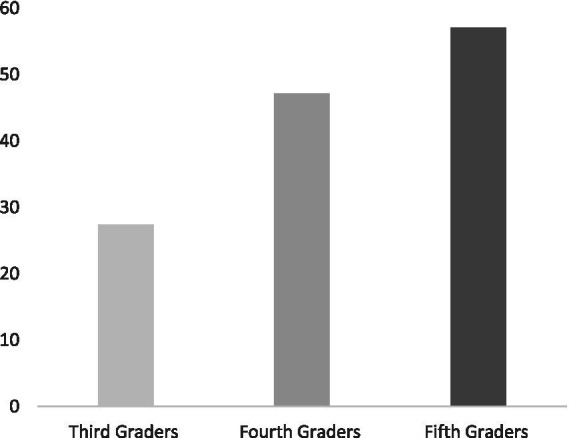
Graphic display of the mean performance on the M-CBM measure reported in correct digits in two minutes.

Tables 1, 2 present the descriptive analyses on the measures including the means, standard deviations, and ranges among third graders, fourth graders, and fifth graders. This descriptive information was helpful in understanding the data and making initial inferences on the differences among all groups. These descriptive statistics also allowed providing visual graphs that facilitated a clear depiction of the data. Figures 2, 3 display the average performance of the Arabiya Calculation Test and M-CBM of the three groups of this study. In general, the preliminary results indicate differences among all groups in the study measures. A closer inspection of the data analyses that addressed the study’s questions is followed.

**Table 1:** Descriptive information of Arabiya calculation test performance in number of correct answers for all groups.

Grade	Number of students	Range	*M*	SD
Third	26	21–26	23.69	1.80
Fourth	26	28–32	30.46	1.67
Fifth	26	33–38	35.96	1.84
All Participants	78	21–38	30.04	5.34

*M, Mean; SD, Standard deviation.*

**Table 2:** Descriptive information of M-CBM performance in CDs for all groups.

Grade	Number of students	Range	*M*	SD
Third	26	22–31	27.36	2.31
Fourth	26	39–50	47.16	2.76
Fifth	26	51–61	57.06	3.29
All Participants	78	22–61	43.86	12.76

CDs, correct digits per two minutes; *M*, mean; SD, standard deviation.

### Internal consistency of the Arabiya calculation test and the M-CBM

The internal consistency of the Arabiya Calculation Test and the Math Curriculum-Based Measurement (M-CBM) were assessed across Grades 3, 4, and 5, with 26 students in each grade. For the Arabiya Calculation Test, Cronbach’s Alpha values were *α* = 0.90 for Grade 3, *α* = 0.89 for Grade 4, and *α* = 0.87 for Grade 5. These results indicate excellent internal consistency, confirming the reliability of the Arabiya Calculation Test across all grades. For the M-CBM, Cronbach’s Alpha values were *α* = 0.85 for Grade 3, *α* = 0.83 for Grade 4, and *α* = 0.87 for Grade 5. These results indicate good to excellent internal consistency, confirming the reliability of the M-CBM across all grades.

### Test–retest reliability

The Arabiya Calculation Test and M-CBM were administered twice to the same sample; the intervening time was one week. The mean scores and standard deviations for the first and second testing and the correlations between the two testing are found in Tables 3, 4. The resulting coefficients, which range from 0.98 to 0.99 for the Arabiya Calculation Test and from 0.82 to 0.99 for M-CBM, are large enough to demonstrate that both measures have high test–retest reliability. In addition, The Standard Error of Measurements (SEMs), reported in Tables 3, 4, can be used to estimate the confidence interval that surround a particular score of the study measures. The SEMs provide an estimate of the variation around a “true” score for an individual when repeated measures are taken. The smaller the SEM, the more confidence one can have in the test’s results. The SEMs of the Arabiya Calculation Test (range from 0.30 to 0.37) and the M-CBM (range from 0.76 to 1.27) were small and suggest a high level of confidence and reliability for the achieved scores.

**Table 3:** Test–retest reliability and SEMs for the Arabiya calculation test using the standard score.

	First testing		Second testing			
Grade	*M*	SD	*M*	SD	*r*	SEMs
Third (*n* = 26)	118.92	2.13	119.00	2.05	0.98	0.30
Fourth (*n* = 26)	119.08	3.52	119.12	3.58	0.99	0.35
Fifth (*n* = 26)	123.23	3.72	123.27	3.60	0.99	0.37
All grades (*n* = 78)	120.41	3.74	120.46	3.70	0.99	0.37

*M, mean; SD, standard deviation; r, correlation coefficient; SEM, standard error of measurement.*

**Table 4:** Test–retest reliability and SEMs for M-CBM.

	First testing		Second testing			
Grade	*M*	SD	*M*	SD	*r*	SEMs
Third (*n* = 26)	27.35	2.30	27.05	2.39	0.89	0.76
Fourth (*n* = 26)	47.15	2.77	47.80	3.20	0.82	1.17
Fifth (*n* = 26)	57.05	3.28	57.40	2.98	0.92	0.92
All Grades (*n* = 78)	43.85	12.75	44.08	13.08	0.99	1.27

*M, mean; SD, standard deviation; r, correlation coefficient; SEM, standard error of measurement.*

### The average differences among the three grades

To explore differences among the three grades, one-way independent Analysis of Variance (ANOVA) was performed. All assumptions of performing ANOVA on the Arabiya Calculation Test raw scores were examined. No violations of normality and homogeneity of variance were detected. The variances were equal for all three groups, *F*(2, 75) = 0.023, *p* = 0.977. There were significant differences among the three groups/grades in terms of their scores, *F*(2, 75) = 310.79, *p* < 0.001. In addition, there was a significant linear trend, *F*(1, 75) = 619.38, *p* < 0.001, indicating that as the grade gets higher, the performance on Arabiya Calculation Test increased proportionately. Similar statistical check of the assumptions was conducted to perform the ANOVA for the M-CBM. No violations of normality and homogeneity of variance were detected. The variances were equal for all three groups, *F*(2, 75) = 1.967, *p* = 0.147. There were significant differences among the three groups/grades in terms of their scores, *F*(2, 75) = 882.30, *p* < 0.001. In addition, there was a significant linear trend, *F*(1, 75) = 1703.46, *p* < 0.001, indicating that as the grade gets higher, the performance on M-CBM increased proportionately.

### The relationship between the Arabiya calculation test and M-CBM

The Arabiya Calculation Test were correlated with the M-CBM scores for all participants and for each grade. All of the coefficients were statistically significant at the *p* < 0.001 level. The magnitude of all correlations was large. The positive correlations coefficients were 0.84, 0.86, 0.89, and 0.93 for third grade, fourth grade, fifth grade, and all grades, respectively.

### Social validity

Evaluations of social validity focus on the satisfaction with the intervention’s outcomes by those who use the intervention. The participants completed a four-item questionnaire in a yes/no format following the completion of the study. Specifically, the gifted students were asked to evaluate the experiences of taking the Arabiya Calculation Test and M-CBM to measure their computational skills. The researchers read to the participants each item on the student questionnaire and asked them to color in a happy face for “yes” or a frowning face for “no.” Results indicated that the students involved in this study were satisfied with the assessment process. 99% of the students believed that the measures may be used with confidence as fast measures to assess their skills. The researchers as well indicated that they liked the experience of using the study measures to screen for gifted students in mathematics.

## Discussion

Accurate screening assessment tools are needed to identify gifted students early and then to use the results to provide appropriate services. In general, this study aimed to investigate whether the Arabiya Calculation Test and M-CBM could be used to measure computation performance, and if they would be valid, reliable, and practical to screen for MGSs. The following sections discuss our study questions.

### Validity findings

Positive results of content validity, including alignment of the specification tables with the test items and the high agreement among referees on the quality these measures add to the field of gifted education assessment, have been detailed in the method section. In addition, the study emphasizes the concurrent validity and the ability of these tools to differentiate gifted students by grade level. By grounding the Arabiya Calculation Test and M-CBM in Jordanian norms and curriculum, while adhering to psychometric standards, the study ensures that these assessments are valid, reliable, and culturally appropriate for identifying and supporting MGSs. This contextualization enhances their cross-cultural validity and underscores their practical application within the Jordanian educational system. Further discussion is provided for the concurrent validity and the ability of these measures to differentiate gifted students according to their grades in the following section. Further discussion is provided for the concurrent validity and the ability of these measures to differentiate gifted students according to their grades in the following section.

#### Implications of the relationship between Arabiya calculation test and M-CBM

Examinations of concurrent validity between the Arabiya Calculation Test and the M-CBM produced interesting results because the relationships were significant and high, specifically when all grades were merged into one analysis because of the increased statistical power. The significant correlation between the M-CBM and the Arabiya Calculation Test aligns with previous research reporting correlations between CBM measures and other standardised assessments (e.g., Helwig et al., 2002; Shapiro et al., 2006). Both achievement tests such as Arabiya Calculation Test, and M-CBM have been shown to be valid and reliable measurements of math computational skills for screening for gifted students in primary grades (Abu-Hamour and Al Hmouz, 2022b; Abu-Hamour et al., 2015; Mather and Abu-Hamour, 2013). Furthermore, the results of this study suggest that both measures have adequate technical characteristics and can be used as universal screening tools to identify gifted students who are far ahead of their classmates in math computational skills.

#### Grades differences

As was confirmed by previous research (e.g., Abu-Hamour, 2018; Marston, 2012; McGowan et al., 2016), the Arabiya Calculation Test and M-CBM are sensitive measures to the student’s development across ages or grades. Significant differences were found among the three groups of gifted students across the three selected grades in terms of their scores in both descriptive and inferential statistics. In addition, there was a significant linear trend indicating that higher the grade, the better the performance of gifted students on the Arabiya Calculation Test and M-CBM. This finding is in line with what has been suggested earlier regarding that CBM develops in a linear fashion from first to eighth grade (e.g., Hosp et al., 2016; Keller-Margulis et al., 2014). The results of this study may be used as preliminary data for Arabic M-CBM development of future norms similar to the international norms (e.g., AIMSweb: www.aimsweb.com, 2022; Easy CBM: Anderson et al., 2014). It is worth documenting as well that the new method of scoring math computational skills (correct digits per two minutes) was sensitive to growth across ages/grades. In addition, this study suggests that the Arabiya Calculation Test is a promising standardised tool to be used for universal screening to identify gifted students who will excel in math computation skills in the primary grades, then provide them with the appropriate intervention/teaching materials.

#### Comparison with international norms

The results of this study demonstrate alignment between the Arabiya Calculation Test, M-CBM, and international standards for identifying gifted students in mathematics. The design of the Arabiya Calculation Test aligns with Jordan’s Ministry of Education curriculum, integrating key mathematical concepts such as addition, subtraction, multiplication, division, percentages, and equations. The test also draws on insights from the WJ IV Tests of Achievement (ACH), an internationally recognized standardized achievement assessment. While distinct in norms and test items, both the Arabiya Calculation Test and the WJ IV emphasize standardized computation skill measurement. Importantly, this study and the WJ IV Manual confirm that gifted students typically achieve a standard score of 116 or higher on the Calculation Test, the internationally accepted cutoff for identifying gifted students. For the M-CBM, findings align closely with international benchmarks for M-CBM. Hosp et al. (2016) reported that students in the 90th percentile achieve 27, 60, and 48 correct digits (CDs) for grades 3, 4, and 5, respectively. In this study, gifted students achieved a mean of 27.36, 47.16, and 57.06 CDs for the same grades, showing comparable performance patterns. Variations may reflect differences in curriculum content, instructional practices, and students’ familiarity with the CBM procedures in general. These comparisons underscore the compatibility of the Arabiya Calculation Test and M-CBM with international norms while also highlighting their adaptation to meet local educational standards.

### Reliability findings

The two measures were investigated by procedural, inter-rater, and test–retest reliabilities. The resulting coefficients were very high for procedural and inter-rater reliabilities. The internal consistency findings further affirm the reliability of these measures. Cronbach’s Alpha values indicated excellent reliability for the Arabiya Calculation Test across Grades 3, 4, and 5, and ranged from good to excellent for the M-CBM at the same grade levels. These results underscore the robustness of both assessments and their effectiveness in identifying MGSs. Although test–retest reliability is high enough as well, a sizable proportion of the variance in scores was attributable to overall mean differences in performance across probes, most likely reflecting differences in difficulty across the probes, the new learning of participants during the one-week period between the first and second testing, and other uncontrolled human factors. Similar findings regarding the reliability of the study measures have been presented in previous research (e.g., Abu-Hamour et al., 2015; Hosp et al., 2016; National Center on Response to Intervention, 2010). In addition, very small SEMs were detected in this study which leads to the conclusion that the Arabiya Calculation Test and M-CBM are consistent across a short period of time and across different examiners and can be administered during the academic year to screen the gifted students and then monitor their progress in math computational skills.

### Limitations, future research, and implications

This study has several limitations to consider. First, data were only collected on third, fourth, and fifth grade gifted students; consequently, the generalizability of findings to other grades or other student populations is unknown. Second, the sample size was relatively small, and all students came from private schools. The sample size of 78 students, equally distributed across grades 3, 4, and 5, was selected from private schools due to the study’s focus on gifted students and the availability of enrichment programs and resources necessary for identifying and supporting MGSs in these schools. While a larger sample would enhance statistical power and generalizability, this was the maximum number achievable within the study’s context. We acknowledge that limiting the sample to private schools restricts the applicability of the findings to public schools and other socio-economic groups. Future research should include diverse school settings and larger samples to improve generalizability. Future research should include diverse school settings and larger samples from Jordan and other Arab countries to improve generalizability. These tools, developed based on the Jordanian curriculum and international assessment standards, require further validation for use in other Arabic-speaking countries. Third, the reliance on teacher nominations as part of the selection criteria may have introduced bias, potentially overlooking students with hidden talents or those who do not conform to conventional characteristics of giftedness. Teacher nominations may favor students with strong academic performance or visible talents, while underestimating creative, culturally diverse, or less outspoken students. Future studies should explore more objective and inclusive screening methods, such as standardized testing or multi-criteria assessment, to mitigate these biases and ensure broader identification of gifted students.

Fourth, additional development and field testing of M-CBM probes are recommended prior to more widespread use of them for absolute decisions (e.g., comparing specific scores to cutoffs, progress monitoring for individual students, or establishing benchmarks). Future research should further compare the instruments with other student populations (e.g., average students, students with dyscalculia). To ensure that identified gifted students receive appropriate support, this study emphasizes the importance of designing targeted intervention strategies and curriculum modifications based on the assessment results. Enrichment programs should focus on advanced mathematical concepts such as algebra, geometry, and problem-solving, introduced through differentiated instruction and inquiry-based learning. Curriculum modifications should promote higher-order thinking skills, including logical reasoning and critical analysis, to challenge gifted learners and foster their potential. In addition, educators should receive specialized training in designing individualized learning plans and employing formative assessments to track progress effectively. Policymakers must allocate resources and establish specialized programs to bridge gaps in gifted education, ensuring that MGSs have access to enriched opportunities that align with their unique abilities and developmental needs. Fifth, both the Arabiya Calculation Test and M-CBM primarily focus on computational skills, potentially overlooking broader mathematical abilities such as problem-solving, logical reasoning, and mathematical reasoning. Future research should incorporate additional tools or develop new measures that evaluate these broader abilities, enabling a more holistic understanding of mathematical potential among gifted students.

The validity findings of Arabiya Calculation Test and M-CBM in the field of gifted education will play a crucial role in informing and guiding practitioners in their decision-making and interventions. Accurate identification of gifted students is ensured through the use of reliable and valid assessment instruments, avoiding misidentification or underrepresentation and enabling appropriate educational provisions for those with exceptional math abilities. In addition, practitioners can confidently plan and implement enrichment programs and acceleration strategies based on the results of the study assessment tools, maximizing the potential and engagement of gifted students. By relying on evidence-based decisions grounded in the validity of assessment tools, practitioners can choose appropriate instructional materials, teaching methods, and grade-level advancements. Furthermore, the validity findings serve as evidence to advocate for resources, funding, and policy changes that support gifted education programs, enabling practitioners to improve support for gifted learners at various levels. Collaboration with researchers and sharing validity data from practical applications contributes to the advancement of the field of gifted education, facilitating continuous improvement in the identification and support of gifted students.

Teachers and school psychologists face the complex task of meeting the diverse needs of students while ensuring progress toward high academic standards. Early identification of gifted students is critical to designing effective interventions based on scientific data. The present study highlights the potential of the Arabiya Calculation Test and M-CBM as valuable tools for screening MGSs. Both assessments have been validated in Western contexts and show promise in Arab countries, such as Jordan. The Arabiya Calculation Test offers a variety of scores (e.g., age equivalents, grade equivalents, standard scores, and percentile ranks) that provide insights into developmental levels, instructional needs, and relative standing in a group. On the other hand, the M-CBM offers a snapshot of current performance and enables the monitoring of progress over time. Together, these tools complement each other by informing diagnostic and instructional decision-making, supporting both general and inclusive education systems (Schneider et al., 2018). Their ease of administration, low cost, and robust validity and reliability further enhance their utility. However, successful implementation of these tools in inclusive educational settings requires addressing practical challenges. One key challenge is ensuring teachers and school psychologists are adequately trained to administer and interpret the tools effectively. Training should focus on enabling educators to understand scoring procedures, accurately identify gifted students, and design instructional strategies based on assessment results. Resource limitations, particularly in underfunded and public schools, also pose barriers. The availability of materials, technological support, and time for training sessions significantly impacts the adoption of these tools. Collaborative efforts with educational authorities can help integrate these tools into existing frameworks and allocate necessary resources.

Cultural considerations are equally critical. Tools like the Arabiya Calculation Test and M-CBM must align with the linguistic and curricular priorities of local educational systems to ensure relevance and acceptance. For instance, the focus on solving mathematical operations should reflect the curriculum and pedagogical approaches used in Jordanian schools. As with all students with special needs, early identification is essential for gifted students. Early screening using tools such as the Arabiya Calculation Test and M-CBM provides a foundation for designing positive and supportive interventions. Gifted students benefit most from enriched educational opportunities when identified early. However, evidence suggests that Jordanian schools are not fully meeting the educational needs of gifted students (Al-Hroub, 2023). Addressing these gaps requires not only the use of effective screening tools but also comprehensive implementation strategies, including teacher professional development, accessible resources, and ongoing research to evaluate their impact in diverse settings. By addressing these challenges and utilizing the strengths of the Arabiya Calculation Test and M-CBM, schools can better support the assessment and educational development of MGSs.

## Data Availability

The raw data supporting the conclusions of this article will be made available by the authors, without undue reservation.
